# Expression of immune response genes in human corneal epithelial cells interacting with *Aspergillus flavus* conidia

**DOI:** 10.1186/s12864-021-08218-5

**Published:** 2022-01-05

**Authors:** Divya Arunachalam, Shruthi Mahalakshmi Ramanathan, Athul Menon, Lekshmi Madhav, Gopalakrishna Ramaswamy, Venkatesh Prajna Namperumalsamy, Lalitha Prajna, Dharmalingam Kuppamuthu

**Affiliations:** 1 Proteomics Department, Aravind Medical Research Foundation, Dr. G. Venkataswamy Eye Research Institute, Aravind Eye Care System, Madurai, Tamil Nadu India; 2 Department of Biotechnology, Alagappa University, Karaikudi, Tamil Nadu India; 3 Theracues Innovations Private Limited, Bangalore, India Karnataka; 4 Cornea clinic, Aravind Eye Hospital, Aravind Eye Care System, Madurai, Tamil Nadu India; 5 Department of Ocular Microbiology, Aravind Eye Hospital, Aravind Eye Care System, Madurai, Tamil Nadu India; 6 Aravind Medical Research Foundation, Dr. G.Venkataswamy Eye Research Institute, Aravind Eye Care System, No.1 Anna Nagar, Madurai, Tamil Nadu India

**Keywords:** Fungal keratitis, *Aspergillus flavus*, Corneal epithelial cells, Immune response, Targeted transcriptomics, NanoString analysis

## Abstract

**Background:**

*Aspergillus flavus*, one of the causative agents of human fungal keratitis, can be phagocytosed by human corneal epithelial (HCE) cells and the conidia containing phagosomes mature into phagolysosomes. But the immunological responses of human corneal epithelial cells interacting with *A. flavus* are not clear. In this study, we report the expression of immune response related genes of HCE cells exposed to *A. flavus* spores using targeted transcriptomics.

**Methods:**

Human corneal epithelial cell line and primary cultures were grown in a six-well plate and used for coculture experiments. Internalization of the conidia was confirmed by immunofluorescence microscopy of the colocalized endosomal markers CD71 and LAMP1. Total RNA was isolated, and the quantity and quality of the isolated RNA were assessed using Qubit and Bioanalyzer. NanoString nCounter platform was used for the analysis of mRNA abundance using the Human Immunology panel. R-package and nSolver software were used for data analysis. KEGG and FunRich 3.1.3 tools were used to analyze the differentially expressed genes.

**Results:**

Different morphotypes of conidia were observed after 6 h of coculture with human corneal epithelial cells and found to be internalized by epithelial cells. NanoString profiling showed more than 20 differentially expressed genes in immortalized human corneal epithelial cell line and more than ten differentially expressed genes in primary corneal epithelial cells. Distinct set of genes were altered in their expression in cell line and primary corneal epithelial cells. KEGG pathway analysis revealed that genes associated with TNF signaling, NF-KB signaling, and Th17 signaling were up-regulated, and genes associated with chemokine signaling and B cell receptor signaling were down regulated. FunRich pathway analysis showed that pathways such as CDC42 signaling, PI3K signaling, and Arf6 trafficking events were activated by the clinical isolates CI1123 and CI1698 in both type of cells.

**Conclusions:**

Combining the transcript analysis data from cell lines and primary cultures, we showed the up regulation of immune defense genes in *A. flavus* infected cells. At the same time, chemokine signaling and B cell signaling pathways are downregulated. The variability in the expression levels in the immortalized cell line and the primary cultures is likely due to the variable epigenetic reprogramming in the immortalized cells and primary cultures in the absence of any changes in the genome. It highlights the importance of using both cell types in host-pathogen interaction studies.

**Supplementary Information:**

The online version contains supplementary material available at 10.1186/s12864-021-08218-5.

## Introduction

*Aspergillus flavus* is the second most common cause of invasive and non-invasive aspergillosis [1, 2]. *A. flavus* produces nonmotile conidia, and under extreme conditions, sclerotia were produced [3, 4]. The conidia are larger in size (3 to 6 μm) than *A. fumigatus* (2 to 3.5 μm), that helps in their deposition in the upper respiratory tract, which could cause fungal sinusitis [5, 6]. In tropical countries *A. flavus* is the predominant pathogen causing fungal keratitis of the cornea [5] along with *Fusarium solani*. Fungal keratitis is the leading cause of ocular morbidity and blindness, especially in tropical countries [7], and it has been estimated that the annual incidence is 113 per 100,000 population in Madurai, South India [8, 9]. Infection occurs predominantly when there is a loss of epithelial integrity owing to trauma or physical injury to the cornea [10]. Treatment options to combat fungal infections are limited, and the only approved drug for topical ophthalmic application for fungal keratitis is natamycin [11]. *In vitro* studies show voriconazole is better than natamycin as an antifungal antibiotic [12], however, in a clinical trial, natamycin is shown to be superior for treating fungal keratitis [13].

Interaction of *A. fumigatus* with macrophages and lung epithelial cells has been studied extensively, and these cells can internalize the fungal spores by phagocytosis. Macrophages internalize about 80% of the *A. fumigatus* conidia, and almost all the conidia are positive for lysosome associated membrane glycoprotein 1 (LAMP1) in the phagosomes at 60 min of infection [14]. Unlike macrophages, roughly 30% of *A. fumigatus* spores are internalized by lung epithelial cell line A549 [15], and roughly half the internalized conidia colocalize with endosomal markers LAMP1 and CD63 after 24 h of infection. Interestingly the internalized conidia survive and germinate inside the host phagosomes by 24 h [16]. The data indicate that the engulfed conidia matured by acquiring late endosomal marker LAMP1 or CD63 in macrophages and epithelial cells. Corneal epithelial cells upregulate the expression of receptors such as dectin-1 [17], TLR-2 and TLR4 [18] upon stimulation with inactivated *A. fumigatus*. Proinflammatory cytokines such as CXCL1, TNF-α, and IL-6 and activation of P38 MAPK are induced through lectin-like oxidized low-density lipoprotein receptor 1 (LOX-1) in corneal epithelial cells mixed with killed *A. fumigatus* [19]. These studies confirm the innate function of human corneal epithelial (HCE) cells towards fungal pathogen through expression and secretion of cytokines, and chemokines.

The transcriptional response of lung epithelial cells against *A. fumigatus* has revealed increased expression of genes involved in innate immune response, chemotaxis, and inflammatory response [20–24]. But the knowledge on the transcriptional response of corneal epithelial cells against fungal infection is not known. We have shown recently that the corneal epithelial cells engulf the *A. flavus* conidia via actin-dependent process [25]. Phagosomes with conidia mature by acquiring early and late endosomal markers, and we also showed the acidification of the endosomes carrying the conidia. In this report, the response of corneal epithelial cells after *A. flavus* infection was studied at the transcriptional level using the immunology panel of the NanoString platform using a coculture experimental setup. The nCounter analysis revealed the differential expression of genes involved in TNF signaling, Th17 differentiation, NF-kB signaling, and B cell receptor signaling. In addition, we observed differences in the gene expression profile between cell line and primary cultures in coculture experiments. Together, the data clearly show that the corneal epithelial cells, an earliest cell type of cornea which encounter the invading pathogen, are the first line of defense against fungal infection and can regulate the host’s innate immune response.

## Methodology

### Cell culture

Simian virus (SV) 40 immortalized Human Corneal Epithelial cell line (RCB2280) was obtained from Riken Cell Bank, Japan, in 2012. The cells were cultured in DMEM/F12, supplemented with 5% FBS, 5 µg/ml insulin, 5 ng/ml human epidermal growth factor, and 0.5% Pencillin-Streptomycin at 37 °C under 95% humidity and 5% CO_2_ as described by Araki Sasaki et al. [26]. Primary Human Corneal Epithelial cells were generated from limbal explants from cadaver eye globes. Human donor’s eyes were obtained from Rotary Aravind International Eye Bank, Madurai, with donor age less than 70 years with no history of ocular trauma and infection. The written informed consent was obtained for the donor eyes from their family. The tissues were handled in accordance with the Tenets of the declaration of Helsinki. The Institutional Review Board of the Aravind Medical Research Foundation, Madurai, approved this study (ID NO. IRB2011001BAS). The cornea was excised from the eye and the limbal explants from the peripheral cornea were placed in the six-well plates and cultured in DMEM/F12 along with hydrocortisone, sodium selenite, insulin, hEGF, gentamicin, amphotericin, DMSO, and transferrin until it reaches confluency [27].

### Preparation of *A. flavus* conidia

Clinical isolates of *A. flavus* were obtained from keratitis patients at Aravind Eye Hospital, Madurai. *A. flavus* (CI1698) is an isolate from ulcer healed patients by anti-fungal therapy (healed case), and *A. flavus* (CI1123) is an isolate that was refractory to antifungal therapy (surgery case). All the fungal cultures were grown in Czapek Dox agar (M075, HiMedia, L.B.S.Marg, Mumbai) plates for seven days at 30 ºC and spores were collected using PBS with 0.05% tween-20. The mycelium from the spore suspension was removed by using a 0.45µ filter (Supor® 450 membrane disc filters, 0.45 μm-47mm, Pall, USA) and spore suspensions were counted using the Neubauer Hemocytometer chamber (Z359629, Merck, Germany). The freshly harvested spores were used for infecting corneal epithelial cells and the remaining spores were aliquoted as 10^8^/ml in 20% glycerol and stored at -80 ºC freezer [28].

### Immunofluorescence analysis

RCB2280 cells were cultured in 22 × 22mm coverslips and mixed with conidia (CI1698) at a multiplicity of ten and incubated for 6 h [25]. Following infection, cells were washed with 1X PBS, fixed with optimized fixation and permeabilization methods for CD71 (4% PFA and 0.1% triton X-100) and LAMP1 (MeOH-Acetone). After blocking, cells were incubated with mouse monoclonal antibodies of CD71 (transferrin receptor) (sc-65882, Santa Cruz Biotechnology, Texas, USA) or LAMP1 (sc-20011, Santa Cruz Biotechnology, Texas, USA) (1:50) for 1 h at room temperature. Finally, the cells were stained with goat anti-mouse IgG conjugated with dylight 550 (1:50) for 1 h at room temperature. Coverslips were mounted on glass slides using vectashield mounting medium containing DAPI and sealed with nail polish. The images were acquired using Leica TCS SP8 confocal laser scanning microscopy and analyzed using LAS AF Lite software.

### RNA preparation

1 × 10^5^ RCB2280 cells were seeded in a six-well plate and were grown up to confluence. Limbal explants from cadaver eye globes were plated in six-well plates and incubated for two weeks to obtain confluent growth. *A. flavus* spores (CI1698 & CI1123) were mixed with the cultured cells at a multiplicity of ten and incubated for six hours in a serum-free medium [21]. At the end of the incubation period, the cells were washed with RNase free 1X PBS and lysed with 350 µl RLT buffer. The lysates were passed through a 20-gauge needle for 5-10 times and stored at -80 ºC freezer. Isolation of RNA from control and infected cells was performed according to the manufacturer’s (74104, Qiagen, Germany) instructions and the DNA from the isolated RNA was removed using TURBO DNA free kit (Invitrogen™ AM1907, California, US) as per the instructions.

### NanoString analysis

RNA integrity was determined using a 2100 Bioanalyzer (Agilent Technologies, Santa Clara, CA) and Qubit high sensitivity assay (Q32855, Thermo Fischer Scientific, Massachusetts, US). mRNA transcript abundance was analyzed from 100 ng of extracted RNA using the NanoString nCounter Human Immunology panel (XT-CSO-HIM2-12 & NAV # 115000062) from two separate experiments (RCB2280 cells and primary cells). The Human Immunology panel consisted of 595 genes (580 well-annotated immune response genes and 15 housekeeping genes). Briefly, 70 µl of hybridization buffer was added to Reporter CodeSet to prepare the master mix. To set up the hybridization reactions, each sample tube contained 8 µl of master mix were mixed with 5 µl of the extracted RNA sample. Capture ProbeSet (2 µl) was added to each tube, and samples were hybridized at 65˚C for 18 h. The hybridized samples were separated using the NanoString nCounter SPRINT automated Profiler. Tripartite complexes (captured probes with targets) were aligned on the microscopic surface of the cartridge and was scanned using Maximum resolution (Max FOV) in the nCounter Digital Analyzer to generate RCC files (as per the sprint manual MAN-10017-08). Raw data were normalized using positive control genes (POS_A, POS_B, POS_C, POS_D, POS_E, POS_F) and the house keeping genes (ABCF1, ALAS1, EEF1G, G6PD, GAPDH, GUSB, HPRT1, OAZ1, POLR1B, POLR2A, PPIA, SDHA, TBP, TUBB, RPL19) using geNorm in the advanced nSolver analysis 4.0 software (MAN-C0019-08), and lowly expressed genes were filtered based on p-value. p-value less than 0.05 were considered as differentially expressed genes (Table S1 and S2) [23].

### Enzyme-linked immunosorbent assay

Human IL-8/CXCL8 DuoSet ELISA kit was used to measure IL-8 concentration in the culture supernatant of RCB2280 cells infected with CI1123. RCB2280 cells were cultured in 12-well plate and infected with live CI1123 conidia for 12 h, 16 h, 20 and 24 h. The conditioned medium was collected, filtered using 0.22-micron filter and centrifuged at 12,000 rpm for 10 min. The supernatant was collected and stored at -80ºC freezer. The concentration of IL-8 was determined as described by the manufacturer. The incubation time with the substrate was optimized and found to be six minutes. Absorbance was measured at 450 nm with reference wavelength of 540 nm using Spectramax microplate reader.

### Data analysis

MA plot for the differentially expressed genes was generated using R-package. Pathway analysis was done using KEGG and FunRich 3.1.3 software tools. Cytoscape 3.8.1 tool was used to create the functional network for the differentially expressed genes. Uniprot was used to analyze the functions of differentially expressed genes. Differentially expressed genes from NanoString data were determined using Student’s t-test (p<0.05).

## Results

### Interaction of *A. flavus* conidia with human corneal epithelial cells

**Fig. 1:**
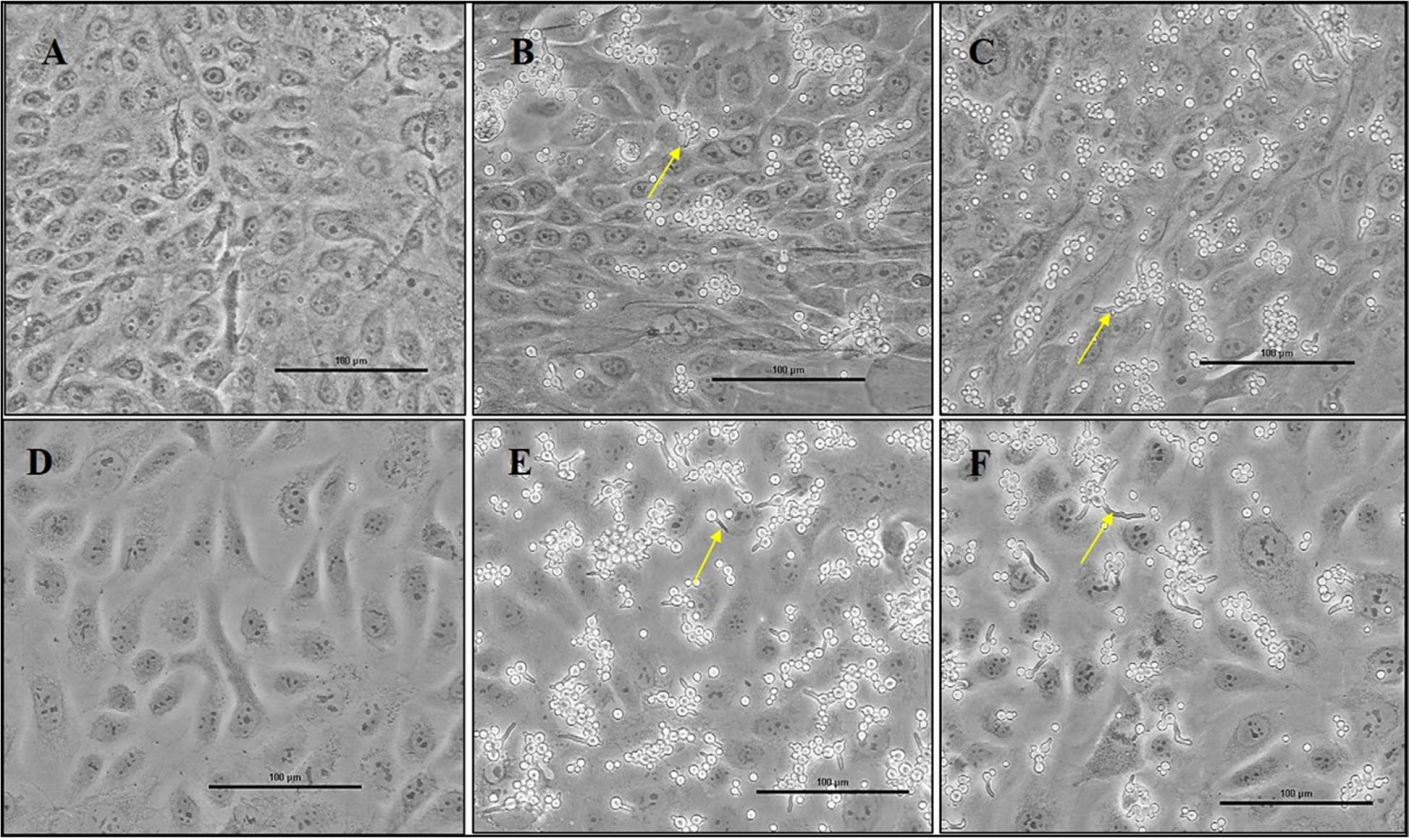
Morphology of uninfected and *A. flavus* infected HCE cells. Microscopic images of primary HCE cells grown on 6-well culture plates were infected with (**A**) no infection (**B**) *A. flavus* CI1698 (**C**) *A. flavus* CI1123 and HCE cell line (RCB2280) infected with (**D**) no infection (E) *A. flavus* CI1698 (**F**) *A. flavus* CI1123 at 200X magnification. The arrow shows the *A. flavus* conidia

Morphotypes of conidia, swollen conidia, and germinated conidia were examined after coculturing the conidia with RCB2280 cells (B&C) or primary cultures (E&F) for 6 h. Clinical isolates 1698 and 1123 were used in these experiments. Almost all the bound conidia were germinated in the cocultures of RCB2280 cells, whereas a smaller number of conidia were germinated in primary HCE cells mixed with conidia (Fig. 1 see arrows). This result implies that the primary cultures can delay the germination of infected conidia.

**Fig. 2:**
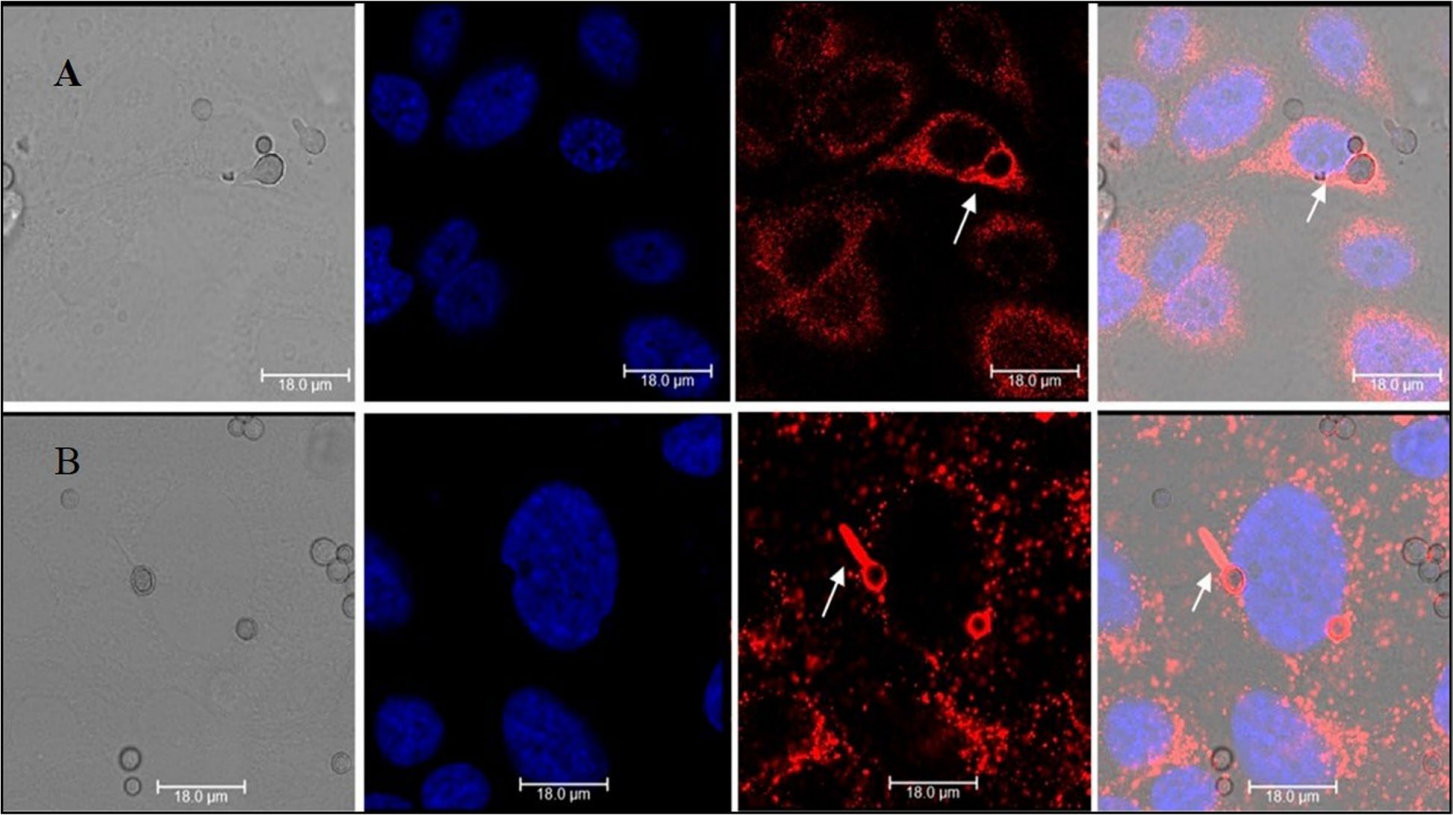
Colocalization of early and late endosomal markers with the *A. flavus* conidia. RCB2280 cells were infected with *A. flavus* (CI1698) swollen spores for 4 h and stained for endosomal markers. (**A**) *A. flavus* in early endosomes (CD71) (**B**) *A. flavus* in late endosomes (LAMP1). The endosomal markers (Red) were detected using dylight 550 conjugated goat anti-mouse secondary antibody. From left to right, panels show bright field, fluorescence image of the blue channel (DAPI), fluorescence image of red channel (endosomes), merged overlay of all fluorescent channels, White arrow shows the germinated conidia inside the early and late endosomes

Further, a fraction of the conidia and germinated conidia were found to be colocalized with the early endosomal marker (CD71) and late endosomal marker (LAMP1) in CI1698 cocultures (Fig. 2). The data indicate that corneal epithelial cells internalized the conidia and the phagosomes containing conidia were matured.

### Differential gene expression of HCE cells upon infection with *A. flavus*

**Fig. 3:**
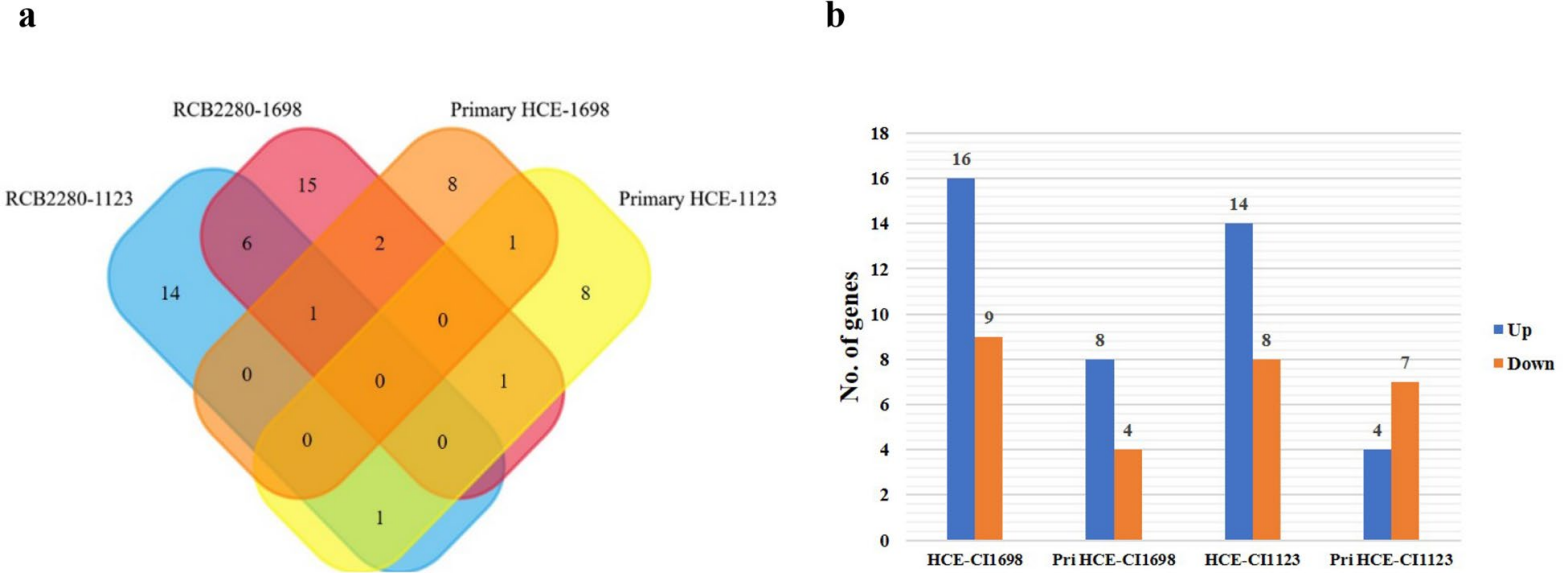
Differential regulation of immune response genes in HCE cells in response to *A. flavus*. (a) Venn diagram shows the comparison of differentially expressed genes between RCB2280 cells and primary HCE cells infected with CI1698 and CI1123. (b) Bar graph for the up and down-regulated genes in RCB2280 cells and primary HCE cells infected with *A. flavus*

Venn diagram shows the overlap of differentially expressed genes of RCB2280 cells and primary cells in response to *A. flavus* infection (Fig. 3a). Expression of six genes, SLC2A1, TNFAIP3, ICAM1, RUNX1, ETS1, and IKZF3 were altered in RCB2280 cells infected with CI1698 and CI1123. On the other hand, transcripts of the LGALS3 gene encoding galectin-3 were the only gene whose expression was up-regulated in primary HCE cells exposed to CI1698 and CI1123. CI1123 induced the expression of KIR activating subgroup 3 genes, while CI1698 induced the expression of PLAUR and ETS1 genes in both the cells. ETS-1 gene was found to be up-regulated in RCB2280 cells cocultured with CI1698 and CI1123 and primary HCE cells infected with CI1698. Around 20 genes were found to be differentially regulated in RCB2280 cells, while the expression of about ten genes were altered in primary cells (Table S3). The number of up-regulated genes was more than the down-regulated genes except in primary HCE cells infected with CI1123 (Fig. 3b).

### Differential gene expression in HCE cells infected with CI1698

**Fig. 4:**
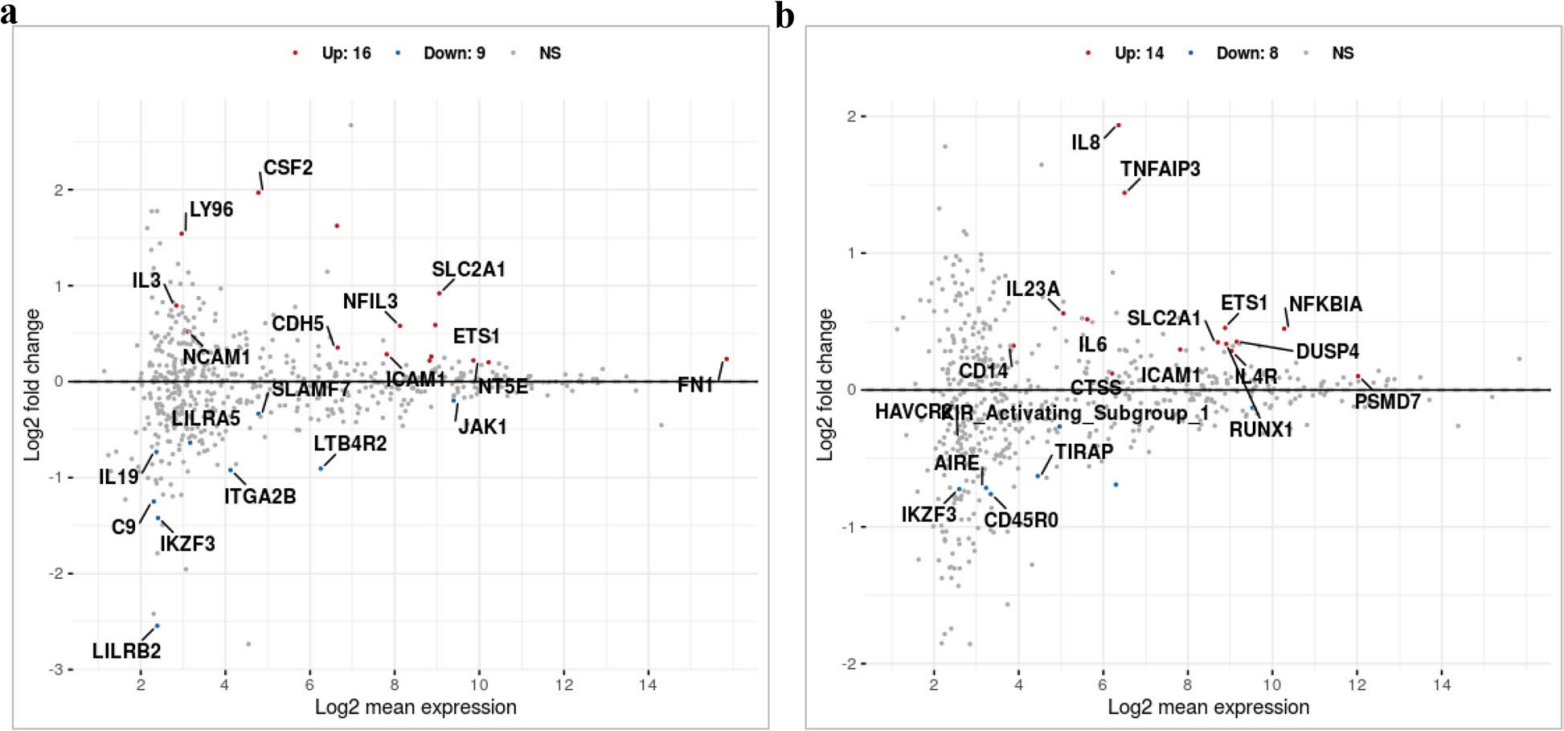
Scatter plot for the differentially expressed genes of HCE cells cocultured with CI1698. MA plot showing the mRNA abundance in (**a**) RCB2280 cells (**b**) primary HCE cells after infection with CI1698 for 6 h. Red dots - Up-regulation; Blue dots – Down-regulation; Grey dots- no change

The MA plot shows the differential mRNA abundance in RCB2280 cells and primary HCE cells infected with CI1698 (Fig. 4a and b). Among the 580 immune response genes quantified, 25 genes were altered in their expression by CI1698 infection of RCB2280 cells, whereas the expression of only 12 genes was altered in primary HCE cells under similar experimental conditions. Of the 25 genes, the expression of 16 genes was upregulated, and nine genes were downregulated in RCB2280 cells with a significant p-value (p<0.05).

**Fig. 5:**
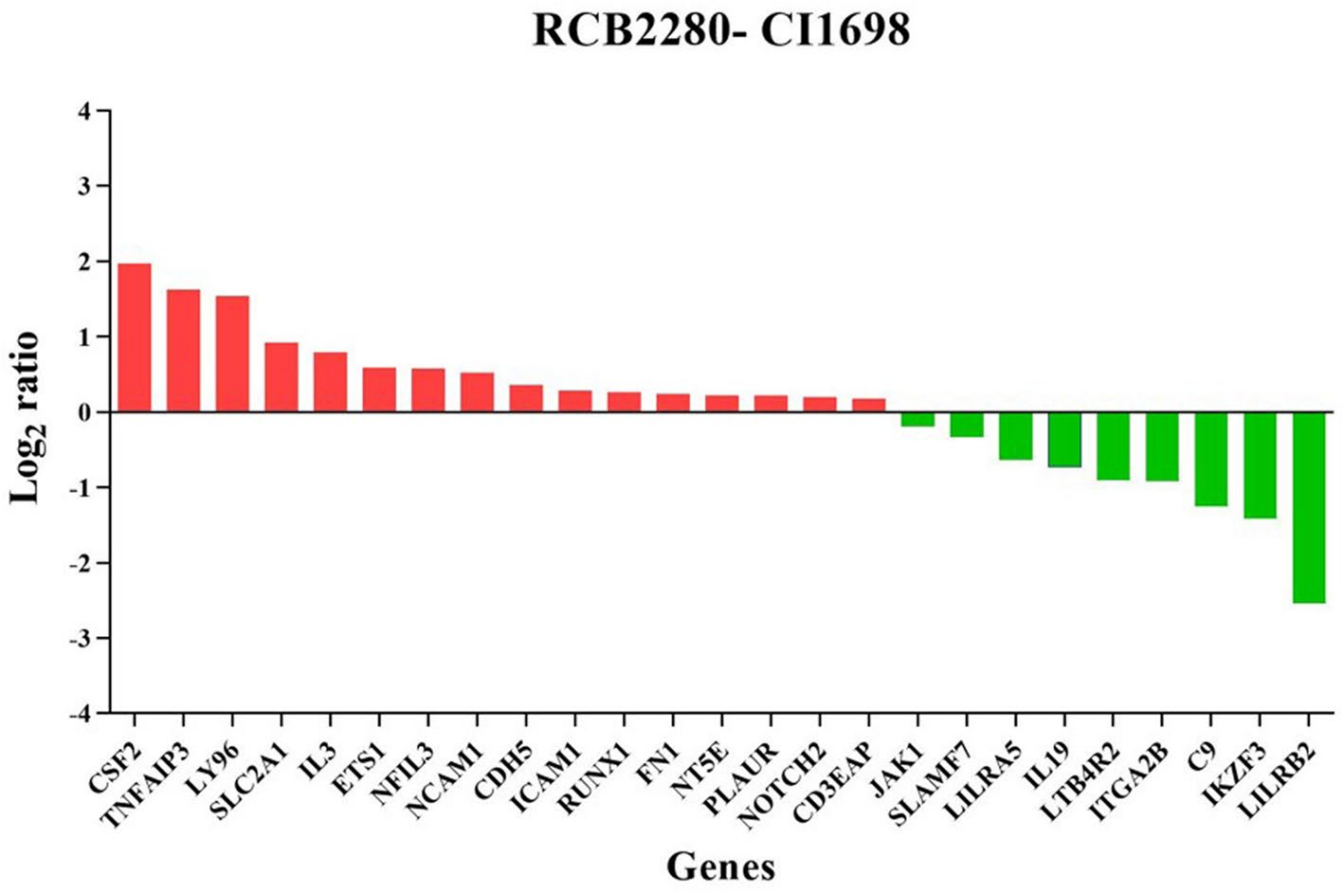
Differentially expressed immune genes of RCB2280 cells exposed with CI1698. Bar graph representing up-regulated (red) and down-regulated (green) genes with corresponding fold change in RCB2280 cells infected with CI1698 for 6 h

**Fig. 6:**
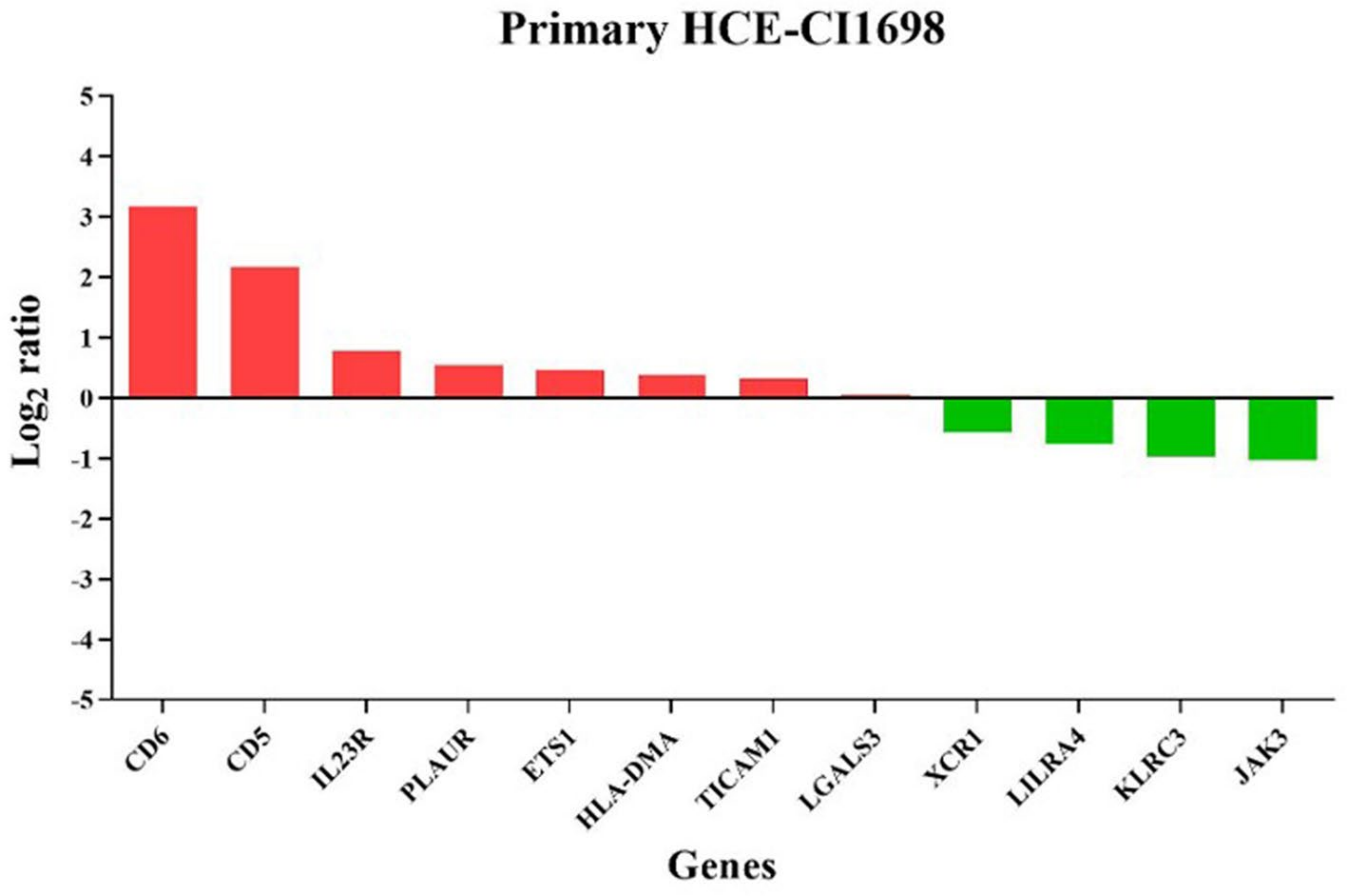
Differentially expressed immune genes of primary HCE cells cocultured with CI1698. Bar graph representing up-regulated (red) and down-regulated (green) genes with corresponding fold change in primary HCE cells infected with CI1698 for 6 h

Figure 5 indicates the differentially expressed genes with corresponding log2 fold change. CSF2 was increased close to log2 fold change of 2, and LILRB2 was reduced with log2 fold change of -2 in RCB2280 cells. In primary HCE cells, eight genes were up-regulated, and four genes were down-regulated compared to uninfected control (Fig. 6). CD5 and CD6 were the ones showing the maximum variation in expression levels in primary cells after infection. The function of differentially expressed genes of RCB2280 cells and primary HCE cells infected with CI1698 is represented in Table 1.

**Table 1:** Functions of differentially expressed genes in HCE cells (cell line and primary) infected with *A. flavus* CI1698

S.No	Gene symbol	Description	Function	Log_2_ Ratio	p value
**Up-regulated genes in RCB2280 cells**					
1	CSF2	Granulocyte-macrophage Colony stimulating factor 2	growth and differentiation of hematopoietic precursor cells	1.9681	0.0262
2	TNFAIP3	Tumor necrosis factor alpha-induced protein 3	Inhibits NF-kB activity	1.6228	0.0455
3	LY96	Lymphocyte antigen 96	Innate immune responses	1.5414	0.0219
4	SLC2A1	Solute carrier family 2, facilitated glucose transporter member 1	Glucose transporter; Facilitate glucose transport to brain	0.9197	0.0042
5	IL3	Interleukin 3	Granulocyte-macrophage colony stimulating factors	0.7921	0.0226
6	ETS1	Protein C-ets-1	Transcription factor; Control expression of cytokines & chemokines	0.5896	0.014
7	NFIL3	Nuclear factor interleukin-3- regulated protein	Transcription regulator; immune response	0.581	0.0075
8	NCAM1	Neural cell adhesion molecule 1	Neuron-neuron adhesion, neurite fasciculation, outgrowth of neurites etc.	0.5205	0.0279
9	CDH5	Cadherin 5	Cell-cell adhesion; cell-cell junction assembly	0.3546	0.0378
10	ICAM1	Intracellular adhesion molecule 1	Transmembrane signaling receptor activity	0.285	0.0164
11	RUNX1	Runt-related transcription factor 1	Attenuator of NF-kB signaling	0.2612	0.0487
12	FN1	Fibronectin 1	Cell adhesion; cell motility; wound healing; cell shape maintenance	0.236	0.019
13	NT5E	5’-nucleotidase	Nucleosidase activity; hydrolyze extracellular nucleotides	0.221	0.0008
14	PLAUR	Urokinase plasminogen activator surface receptor	Promote plasmin formation	0.2182	0.0428
15	NOTCH2	Neurogenic locus notch homolog protein 2	Regulate cell-fate determination	0.2014	0.044
16	CD3EAP	DNA-directed RNA polymerase I subunit RPA34	DNA-directed 5’-3’ RNA polymerase activity; RNA binding	0.1774	0.0483
**Up-regulated genes in primary HCE cells**					
1	CD6	T-cell differentiation antigen CD6	Regulate inflammatory responses; secretion of proinflammatory cytokines to LPS	3.1751	0.0044
2	CD5	CD5 antigen like	Regulate inflammatory responses	2.174	0.0093
3	IL23R	Interleukin 23 R	Mediates stimulation of T-cells, NK-cells, macrophages via JAK-STAT signaling	0.7777	0.0125
4	PLAUR	Urokinase plasminogen activator surface receptor	Promote plasmin formation	0.5552	0.0354
5	ETS1	Protein C-ets-1	Transcription factor; Control expression of cytokines & chemokines	0.4657	0.0471
6	HLA-DMA	HLA class II histocompatibility antigen, DM alpha chain	Antigen processing and presentation	0.3909	0.0347
7	TICAM1	TIR-domain containing adapter molecule 1	TLR signaling	0.3236	0.0134
8	LGALS3	Galectin 3	Ligand for galactose. Innate immune response	0.0466	0.048
**Down-regulated genes in RCB2280 cells**					
1	JAK1	Tyrosine protein kinase 1	IFN-alpha/beta/gamma signaling pathway	-0.1963	0.042
2	SLAMF7	SLAM family member 7	Modulate activation and differentiation of various immune cells	-0.3343	0.0348
3	LILRA5	Leukocyte immunoglobulin-like receptor subfamily A member 5	Triggering innate immune responses	-0.6356	0.0194
4	IL19	Interleukin 19	Inflammatory responses	-0.7323	0.009
5	LTB4R2	Leukotriene B4 receptor 2	Receptor for leukotrienes; mediates chemotaxis	-0.9056	0.0358
6	ITGA2B	Integrin alpha-IIb	Integrin mediated signaling	-0.9215	0.0124
7	C9	Complement component 9	Regulation of complement activation	-1.2485	0.0241
8	IKZF3	Zinc finger protein aiolos	Regulation of lymphocyte differentiation	-1.4196	0.0209
9	LILRB2	Leukocyte immunoglobulin-like receptor subfamily B member 2	Down regulation of immune responses and development of tolerance	-2.5436	0.027
**Down-regulated genes in primary HCE cells**					
1	XCR1	Chemokine XC receptor 1	Calcium mediated signaling; immune and inflammatory responses	-0.5621	0.0201
2	LILRA4	Leukocyte immunoglobulin-like receptor subfamily A member 4	Triggering innate immune responses	-0.7656	0.0259
3	KLRC3	NKG2-E type II integral membrane protein	Receptor for recognition of MHC class I molecules by NK cells and Tc cells	-0.9674	0.033
4	JAK3	Tyrosine protein kinase 3	Mediates signaling event in both innate and adaptive immunity	-1.0351	0.0199

**Fig. 7:**
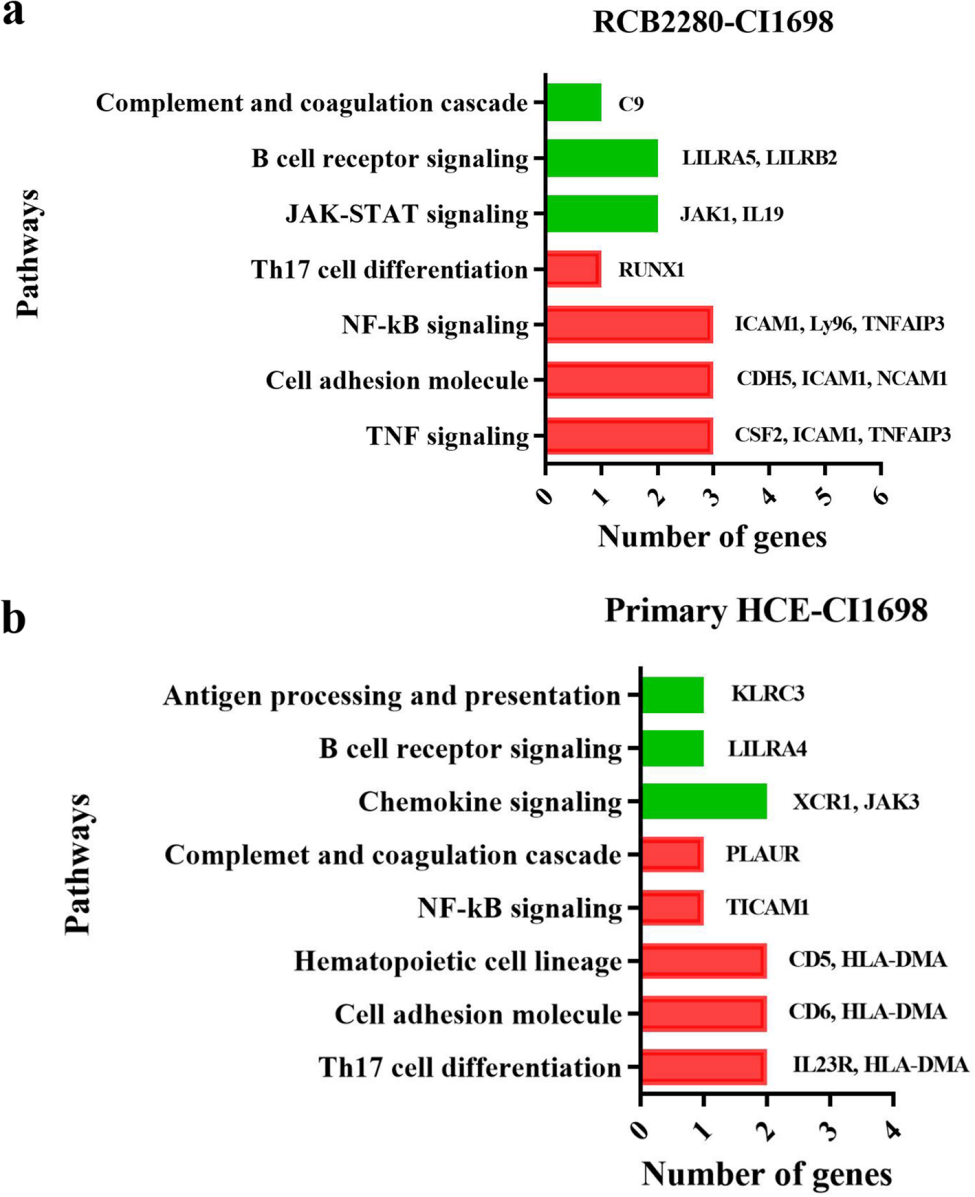
KEGG pathway analysis of up and down-regulated genes of HCE cells infected with CI1698. (**a**) RCB2280 cells infected with CI1698 for 6 h. (**b**) Primary HCE cells infected with CI1698 for 6 h. Red bar - pathways associated with up-regulated genes; Green bar - pathways associated with down-regulated genes

Pathway analysis using KEGG showed the upregulation of genes involved in TNF signaling, cell adhesion pathway, NF-kB signaling, and Th17 differentiation in the coculture of RCB2280 and conidia of CI1698, whereas multiple genes involved in the B cell receptor signaling and JAK-STAT signaling were downregulated (Fig. 7a). In the coculture experiments using primary cultures and CI1698, hematopoietic cells, the lineage pathway is also upregulated, and chemokine signaling is downregulated along with other pathways (Fig. 7b). The experiment using primary culture or RCB2280, infected with CI1698, show similar pattern of alteration in gene expression profile.

**Fig. 8:**
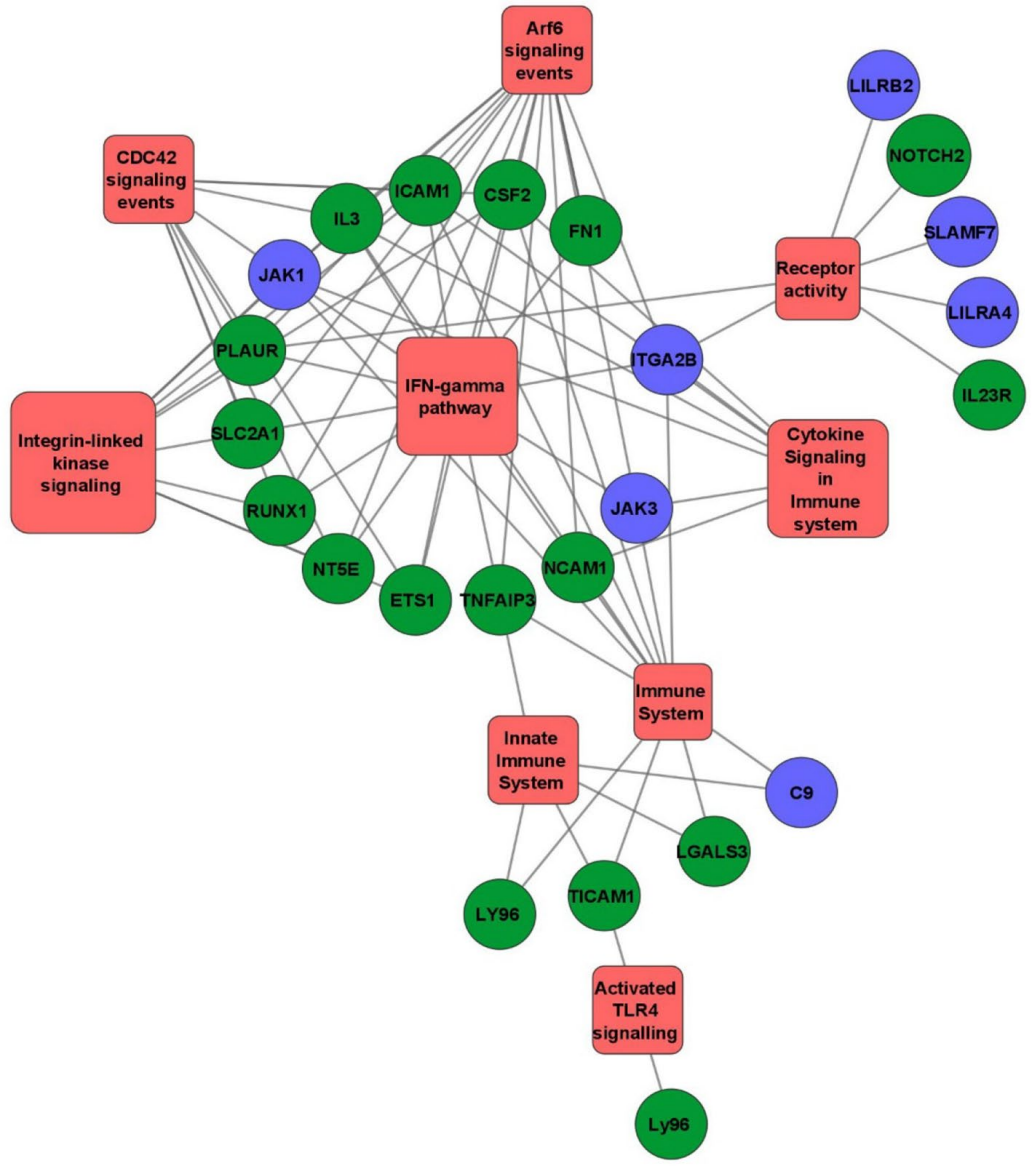
The functionally grouped network of Gene Ontology enrichment analysis. Differentially expressed mRNA transcripts of RCB2280 cells and primary HCE cells infected with CI1698 using FunRich 3.1.3 and Cytoscape 3.8.1 tool. Green circle - RCB2280 cells infected with CI1698; Blue circle - primary HCE cells infected with CI1698; Red rectangle - Biological pathways

Functionally grouped network of Gene Ontology enrichment analysis using FunRich 3.1.3 tool showed pathways enriched in the cocultures of RCB2280 and primary cultures. The number of genes in the enriched pathways is much higher in RCB2280 cell line cocultures (Fig. 8).

## Differential gene expression in HCE cells infected with CI1123

**Fig. 9:**
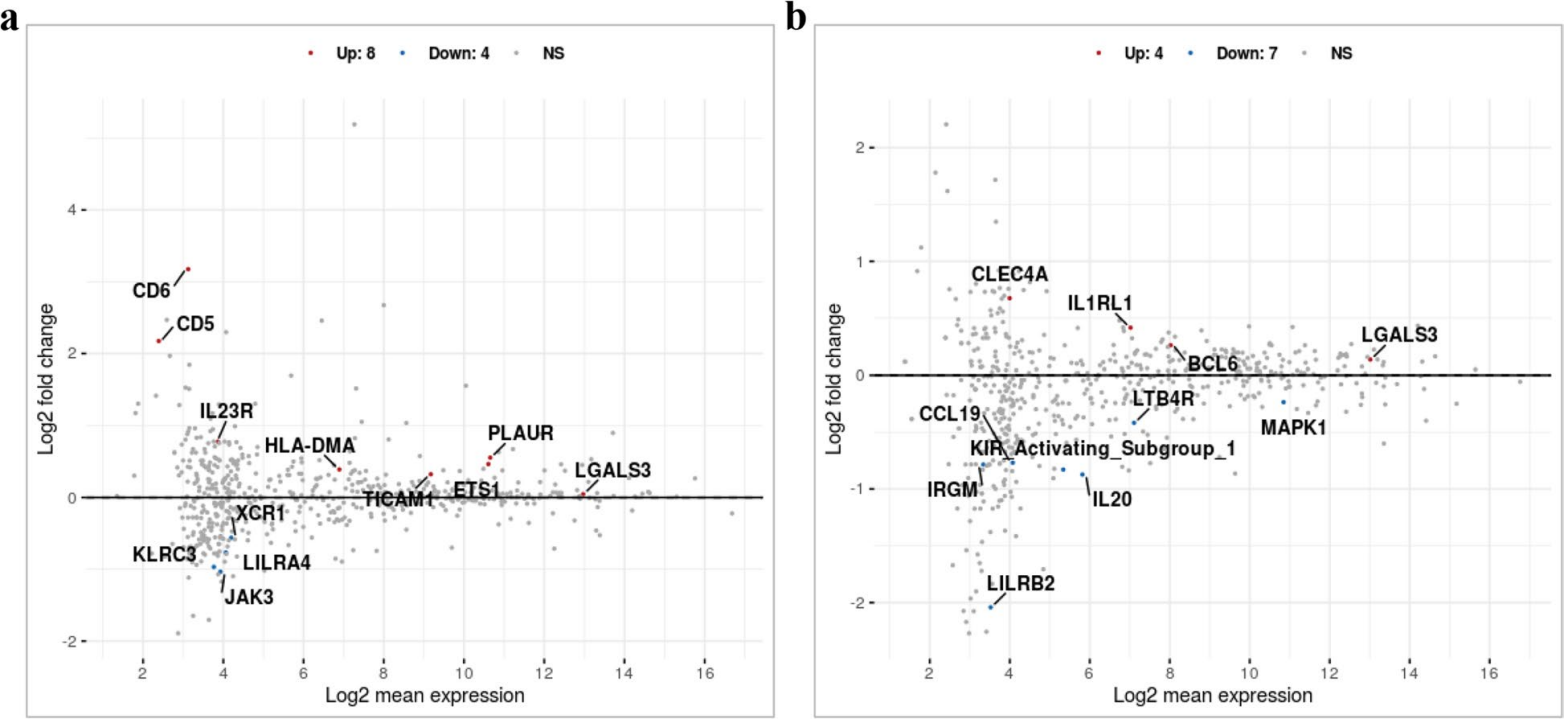
Scatter plot for the differentially expressed genes of HCE cells cocultured with CI1123. MA plot showing the mRNA abundance in (**a**) RCB2280 cells (**b**) primary HCE cells after infection with CI1123 for 6 h. Red dots - Up-regulation; Blue dots – Down-regulation; Grey dots - no change

Figure 9 shows the MA plot for the differential mRNA transcripts for HCE cells in response to CI1123. Only 22 genes and 11 genes were differentially expressed in RCB2280 cells (Fig. 9a) and primary HCE cells (Fig. 9b), respectively. In RCB2280 cells, the expression of 14 genes was increased, and eight genes showed decreased expression compared to the control. In contrast, in primary HCE cells, the expression of only four genes was increased, and the expression of seven genes was decreased.

**Fig. 10:**
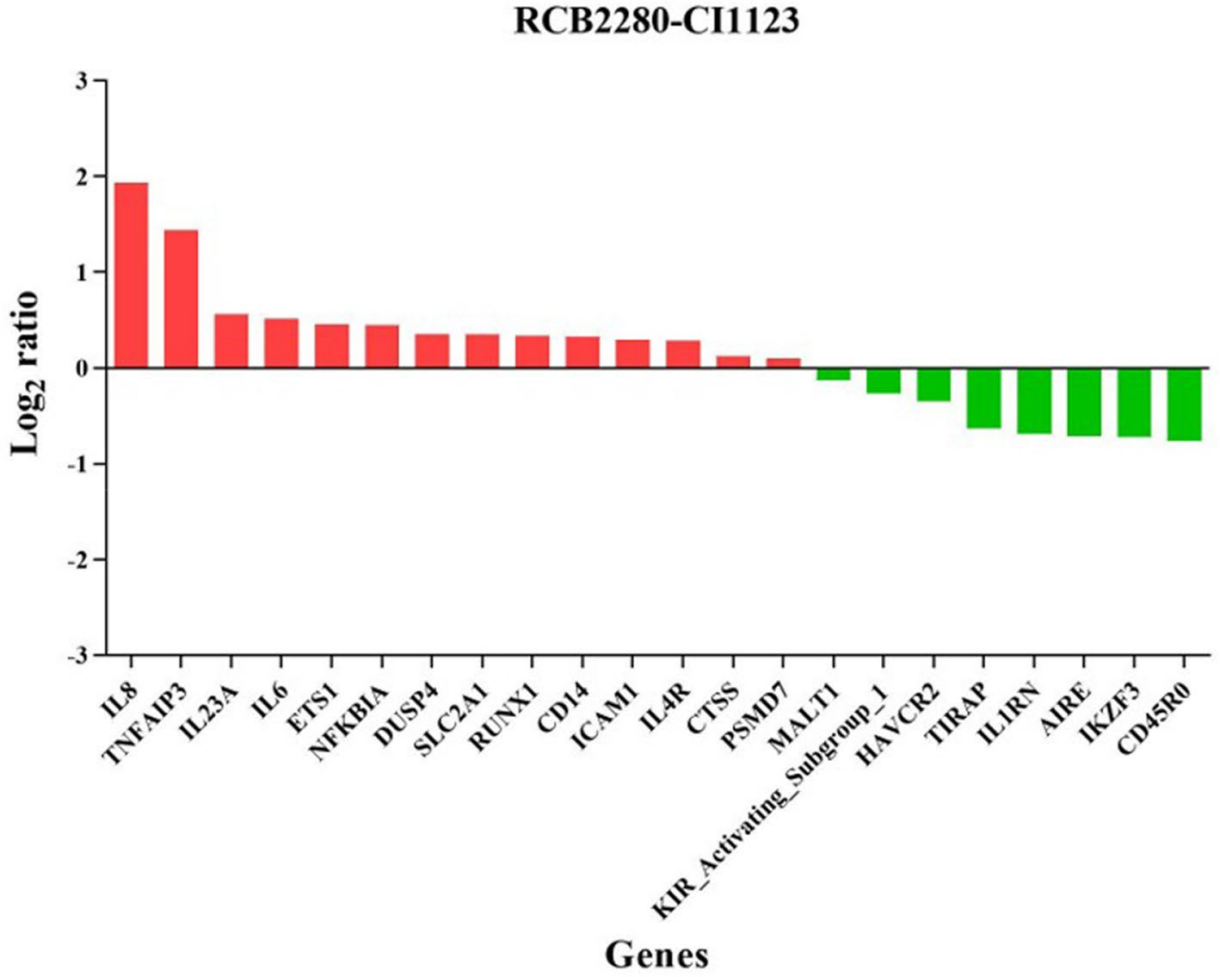
Differentially expressed immune genes of RCB2280 cells exposed with CI1123. Bar graph representing up-regulated (red) and down-regulated (green) genes with corresponding fold change in RCB2280 cells infected with CI1123 for 6 h

**Fig. 11:**
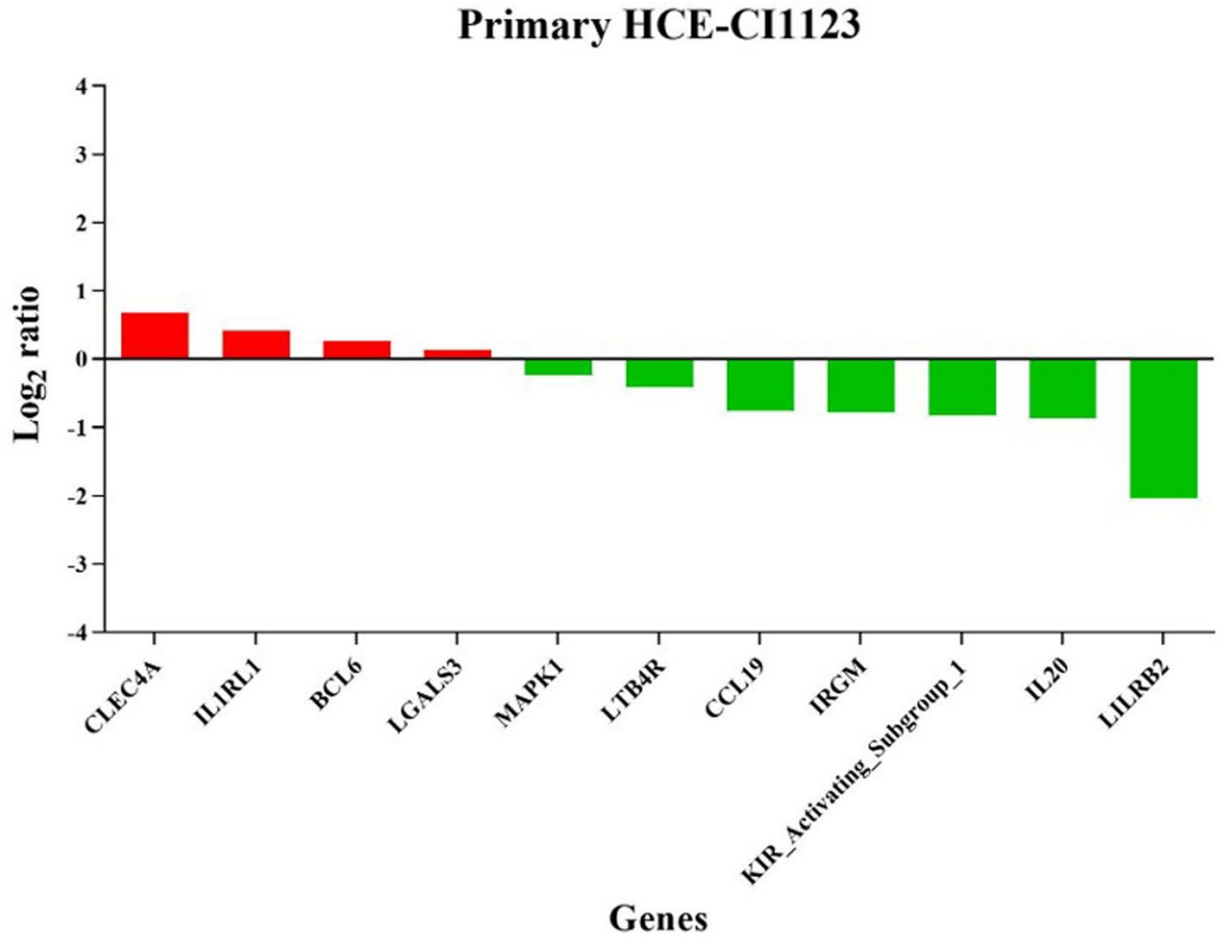
Differentially expressed immune genes of primary HCE cells cocultured with CI1123. Bar graph showing up-regulated (red) and down-regulated (green) genes with corresponding fold change in primary HCE cells infected with CI1123 for 6 h

**Fig. 12:**
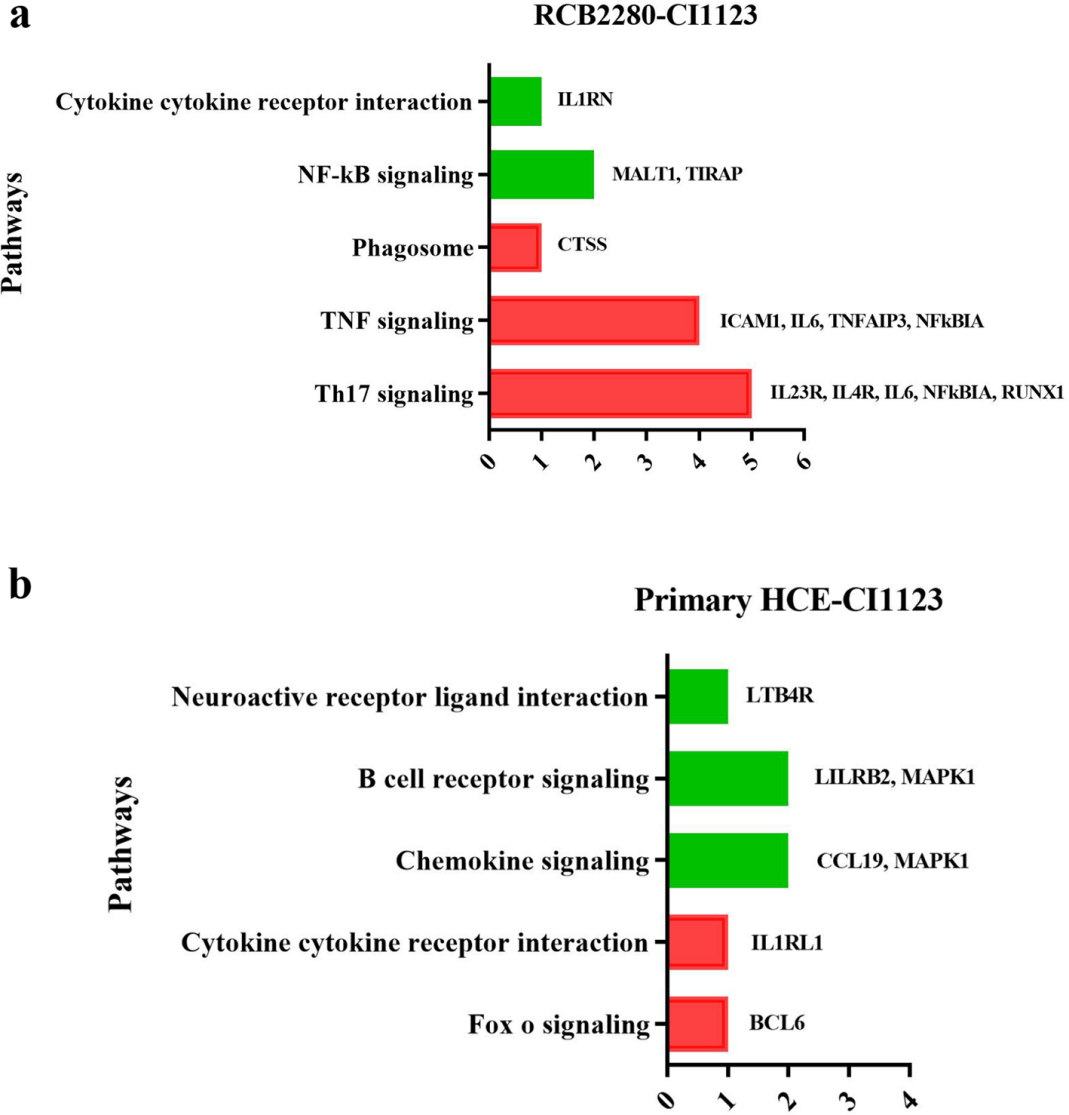
KEGG pathway analysis of up and down-regulated genes of HCE cells infected with CI1123 for 6 h. (**a**) RCB2280 cells infected with CI1123. (**b**) Primary HCE cells infected with CI1123. Red bar - pathways associated with up-regulated genes; Green bar - pathways associated with down-regulated genes

Figures 10 and 11 show the expression level of differentially regulated genes in RCB2280 cells and primary HCE cells. IL8 was the top up-regulated gene in RCB2280 cells, and LILRB2 was a top down-regulated gene in primary HCE cells. The functions of differentially expressed genes of RCB2280 cells and primary HCE cells infected with CI1123 is shown in Table 2. KEGG pathway analysis revealed that the up-regulated genes were involved in Th17 signaling and TNF signaling, and down-regulated genes were involved in NF-kB signaling in RCB2280 cells (Fig. 12a). In primary HCE cells, up-regulated genes were involved in cytokine-cytokine receptor interaction, and down-regulated genes were involved in B cell receptor signaling and chemokine signaling (Fig. 12b).

**Table 2:** Functions of differentially expressed genes in HCE cells (cell line and primary) infected with *A. flavus* CI1123

S.No	Gene symbol	Description	Function	Log_2_ Ratio	p value
**Up-regulated genes in RCB2280 cells**					
1	IL8	Interleukin 8	Chemotactic factor that attracts neutrophils, basophils and T-cells. It is released from several cell types upon inflammatory stimulus	1.93500	0.0372
2	TNFAIP3	Tumor necrosis factor alpha-induced protein 3	Inhibits NF-kB activity	1.44080	0.0441
3	IL23A	Interleukin 23 A	Involve in innate and adaptive immunity; IL17 signaling	0.56002	0.0033
4	IL6	Interleukin 6	Inducer of acute phase response; lymphocyte, monocyte differentiation	0.51664	0.0276
5	ETS1	Protein C-ets-1	Transcription factor; Control expression of cytokines & chemokines	0.45335	0.0231
6	NFKBIA	NF-kappa B inhibitor alpha	Inhibits nucleus translocation of NF-kB/Rel complexes	0.44790	0.0051
7	DUSP4	Dual specificity protein phosphatase 4	Regulates mitogenic signal transduction	0.35252	0.0189
8	SLC2A1	Solute carrier family 2, facilitated glucose transporter member 1	Glucose transporter; Facilitate glucose transport to brain	0.34897	0.0437
9	RUNX1	Runt-related transcription factor 1	Attenuator of NF-kB signaling	0.33727	0.0074
10	CD14	Monocyte differentiation antigen CD14	Innate immune response to bacterial LPS	0.32269	0.0433
11	ICAM1	Intracellular adhesion molecule 1	Transmembrane signaling receptor activity	0.29601	0.0193
12	IL4R	Interleukin 4 receptor	Receptor for IL-4 and IL-13; chemokine production	0.28525	0.0355
13	CTSS	Cathepsin S	Maturation of phagosomes	0.12044	0.0231
14	PSMD7	26 S proteasome non-ATPase regulatory subunit 7	Degradation of ubiquitinated proteins	0.10129	0.0277
**Up-regulated genes in primary HCE cells**					
1	CLEC4A	C-type lectin domain family 4 member A	Binds to mannose and fucose; regulate immune reactivity	0.675	0.0303
2	IL1RL1	Interleukin 1 receptor-like 1	Receptor for IL-33; Involve in helper T-cell function	0.4157	0.0296
3	BCL6	B-cell lymphoma 6 protein	Transcriptional repressor mainly required for germinal center (GC) formation and antibody affinity maturation	0.2613	0.0362
4	LGALS3	Galectin 3	Ligand for galactose. Innate immune response	0.1357	0.006
**Down-regulated genes in RCB2280 cells**					
1	MALT1	Mucosa-associated lymphoid tissue lymphoma translocation protein 1	Enhances BCL10 induced activation of NF-kB	-0.1295	0.0486
2	KIR_Activating_ Subgroup_1	Killer-cell immunoglobulin-like receptor activating subgroup I	Suppress cytotoxic activity of NK cells	-0.2670	0.0107
3	HAVCR2	Hepatitis A virus cellular receptor 2	Modulate innate and adaptive immune response	-0.3465	0.0210
4	TIRAP	Toll/interleukin-1 receptor domain-containing adapter protein	Adapter involved in TLR-2 & TLR4 signaling pathways	-0.6289	0.0251
5	IL1RN	Interleukin 1 receptor antagonist protein	Inhibits activity of interleukin 1	-0.6912	0.0456
6	AIRE	Autoimmune regulator	Transcription factor; promote self-tolerance in thymus	-0.7154	0.0038
7	IKZF3	Zinc finger protein aiolos	Regulation of lymphocyte differentiation	-0.7239	0.0202
8	CD45R0	Receptor type tyrosine protein phosphatase C	T cell activation	-0.7606	0.0452
**Down-regulated genes in primary HCE cells**					
1	MAPK1	Mitogen activated protein kinase 1	cell growth, adhesion, survival and differentiation	-0.2363	0.0038
2	LTB4R	Leukotriene B4 receptor 1	Chemoattractant	-0.4182	0.0314
3	CCL19	C-C motif chemokine 19	Chemotactic activity for T cells and B cells	-0.7688	0.0108
4	IRGM	Immunity-related GTPase family M protein	Regulate autophagy and proinflammatory cytokine production	-0.7853	0.0258
5	KIR_Activating_ subgroup I	Killer-cell immunoglobulin-like receptor activating subgroup I	Suppress cytotoxic activity of NK cells	-0.8292	0.0249
6	IL20	Interleukin 20	Proinflammatory and angiogenic cytokine	-0.873	0.047
7	LILRB2	Leukocyte immunoglobulin-like receptor subfamily B member 2	Down regulation of immune responses and development of tolerance	-2.0416	0.0376

**Fig. 13:**
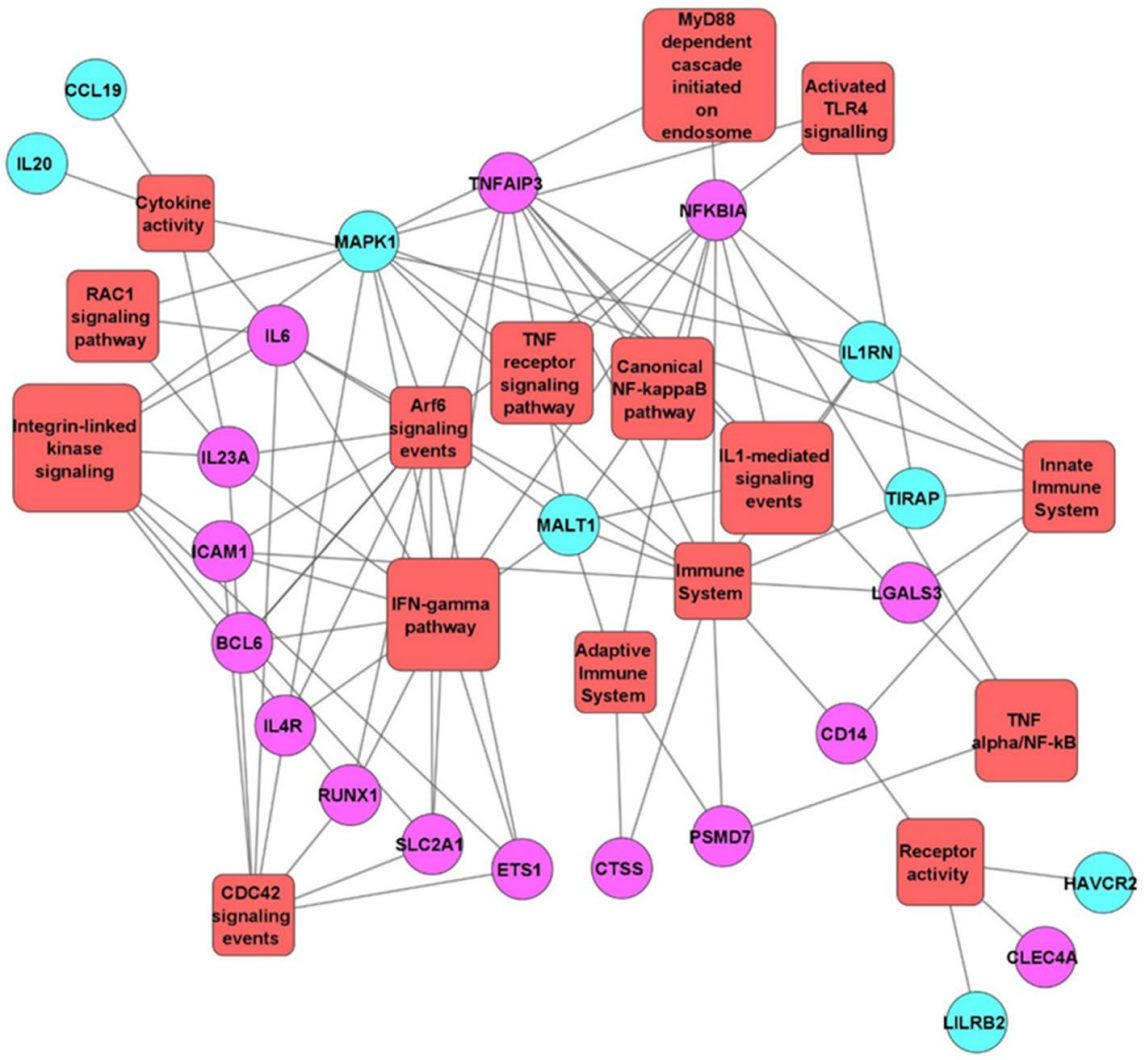
The functionally grouped network of Gene Ontology enrichment analysis. Differentially expressed mRNA transcripts of RCB2280 cells and primary HCE cells infected with CI1123 using FunRich 3.1.3 and Cytoscape 3.8.1 tool. Purple circle - RCB2280 cells infected with CI1698; Blue circle - primary HCE cells infected with CI1123; Red rectangle -Biological pathways

Functionally grouped network of Gene Ontology enrichment analysis of differentially expressed mRNA transcripts using FunRich 3.1.3, and Cytoscape 3.8.1 tool was represented in Fig. 13. The differentially expressed mRNA transcripts of RCB2280 cells were enriched in CDC42 signaling, Arf6 trafficking events, and IFN gamma pathway, and primary HCE cells were enriched in innate immune system and cytokine activity.

### Response of HCE cells to *A. flavus* clinical isolates

**Fig. 14:**
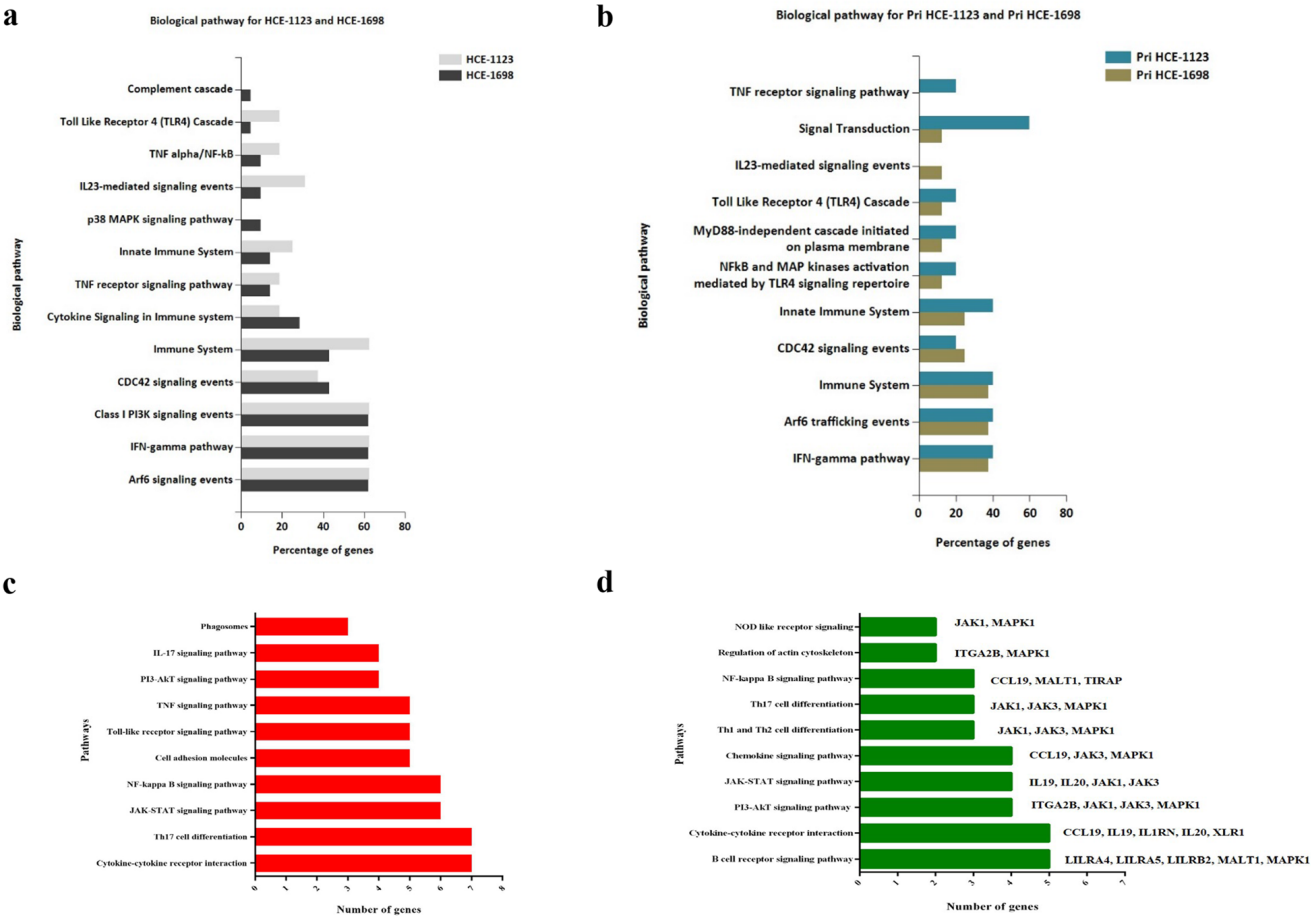
Pathway analysis of HCE cells in response to *A. flavus* clinical isolates. (**a**) Biological pathways of RCB2280 cells infected with CI1698 and CI1123 for 6 h. Grey bar - RCB2280 cells infected with CI1123; Black bar - RCB2280 cells infected with CI1698. (**b**) Biological pathways of primary HCE cells infected with CI1698 and CI1123. Dark green bar - Primary HCE cells infected with CI1123; Light green bar - Primary HCE cells infected with CI1698. (**c**) Up-regulated immune genes associated pathways in HCE cells infected with *A. flavus* clinical isolates. (**d**) Down-regulated immune genes associated pathways in HCE cells infected with *A. flavus* clinical isolates

CI1698 or CI1123 commonly altered eight genes in HCE cells (cell line and primary HCE cells), and the function of these genes is shown in Table 3. FunRich 3.1.3 pathway analysis showed that pathways such as CDC42 signaling, PI3K signaling, and Arf6 trafficking events were equally activated by CI1123 and CI1698 in RCB2280 cells and primary HCE cells. Cytokine-related pathways such as TNF-α/NF-kB, IL23, and TLR 4 signaling were overrepresented in CI1123 infected RCB2280 cells. Similarly, CI1123 infection activates TLR4 signaling and TNF signaling in primary HCE cells, Whereas CI1698 activated complement cascade and p38 MAPK pathway in RCB2280 cells and IL23 mediated signaling in primary HCE cells (Fig. 14a and b). KEGG pathway analysis shows that upregulated genes in response to CI1698 and CI1123 were associated with cytokine-cytokine receptor interaction, TNF signaling, NF-kB signaling pathway, JAK-STAT signaling pathway, Th17 differentiation, and down-regulated genes were associated with chemokine signaling, B-cell receptor signaling pathway, Th1, and Th2 cell differentiation, etc. (Fig. 14c and d).

**Table 3:** Commonly altered genes in HCE cells (cell line and primary) by CI1698 and CI1123

S. No	Genes	Description	Function
1	TNFAIP3	Tumor necrosis factor alpha-induced protein 3	Inhibits NF-kB activity
2	ETS1	Protein C-ets-1	Transcription factor; Control expression of cytokines & chemokines
3	SLC2A1	Solute carrier family 2, facilitated glucose transporter member 1	Glucose transporter; Facilitate glucose transport to brain
4	RUNX1	Runt-related transcription factor 1	Attenuator of NF-kB signaling
5	ICAM1	Intracellular adhesion molecule 1	Transmembrane signaling receptor activity
6	LGALS3	Galectin 3	Ligand for galactose. Innate immune response
7	IKZF3	Zinc finger protein aiolos	Regulation of lymphocyte differentiation
8	LILRB2	Leukocyte immunoglobulin-like receptor subfamily B member 2	Down regulation of immune responses and development of tolerance

### Validation of upregulation of IL-8 expression by ELISA

**Fig. 15:**
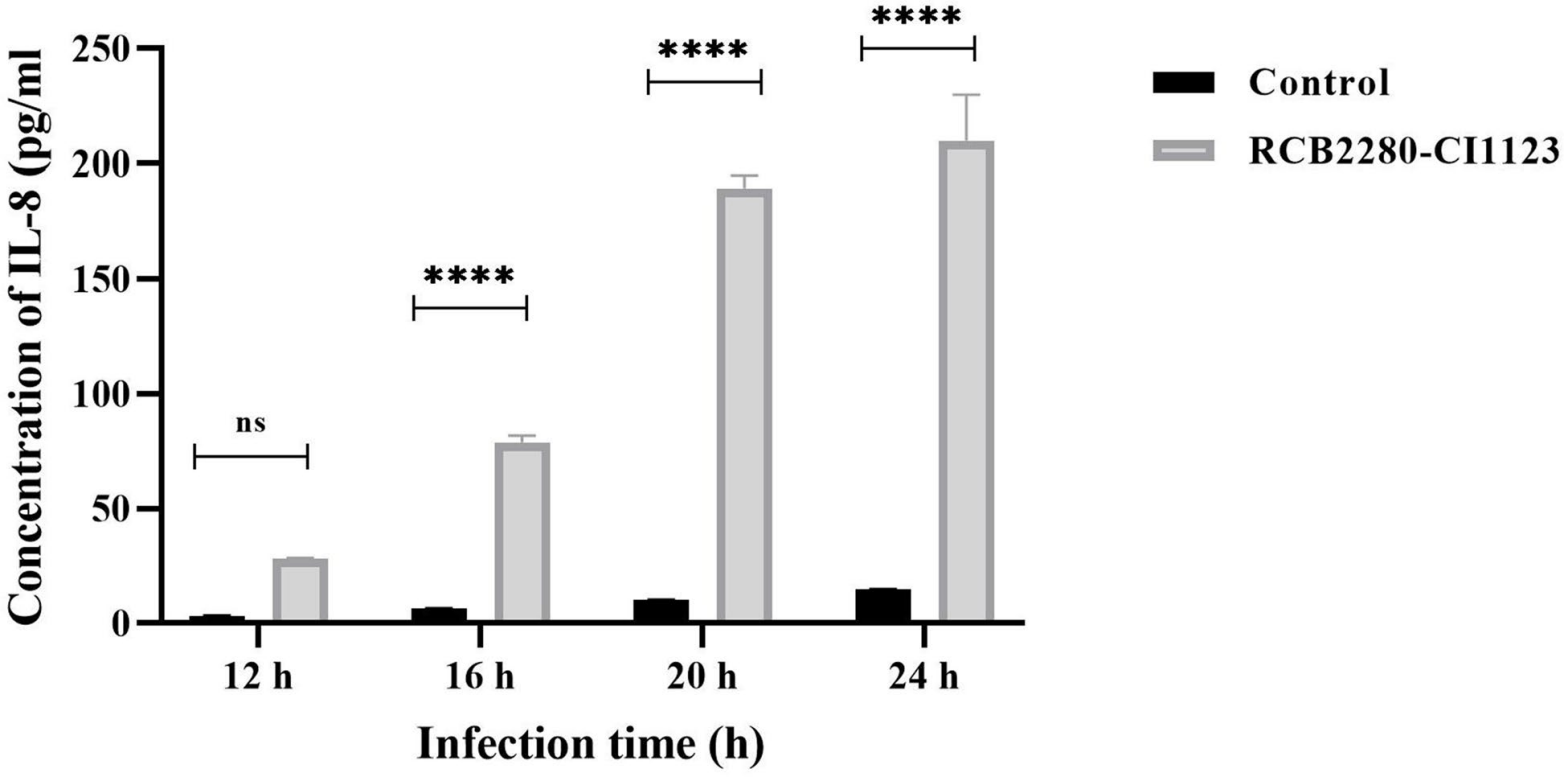
Expression of IL-8 in RCB2280 cells infected with CI1123. RCB2280 cells were cultured in 12-well plate till confluency and infected with CI1123 for 12 h, 16 h, 20h and 24 h. Bar graph representing the concentration of IL-8 at different time point. Black bar -Control; Grey bar - RCB2280 cells infected with CI1123. The data were analyzed using two-way ANOVA with Bonferroni multiple comparison test (ns-no significance; **** p<0.0001)

IL-8 expression was up-regulated in RCB2280 cells infected with CI1123 at the transcript level as shown above. To confirm this result, Human IL-8/CXCL8 DuoSet ELISA kit was used to determine IL-8 protein concentration in CI1123 infected RCB2280 cells. A time dependent increase in the IL-8 amount was observed. IL-8 was elevated from 12 h and reached maximum at 24 h (Fig. 15). IL-8 level was increased by ten-fold in infected RCB2280 cells compared to uninfected control. This data confirms the transcript data and indicates that the mRNA level and protein level are comparable in the infected cells.

## Discussion

Our present study shows that the targeted, relevant transcriptome response of human corneal epithelial cells exposed to clinical isolates of *A. flavus* is not identical in the cell line and primary cultures. At six hours post-infection, different morphotypes of *A. flavus* such as conidia and germinated conidia were observed internal and external to the corneal epithelial cells; however, we found more germinated conidia in the coculture of RCB2280 cells with *A. flavus* compared to a similar experiment using primary cultures. The reduction in the germination in primary cells compared to cell line is not due to the multiplicity of infection since the MOI was kept constant in both cases. The defense pathways specifically activated in the primary cells are the likely cause of the inhibition of germination. It is also important to note that the conidia induce the activation of host response either by internalization or due to the binding of the conidia to the surface receptors. Additional experiments are needed to clarify this. Studies have shown that the internalized *A. fumigatus* spores survive inside lung epithelial cells due to phagosome maturation failure and the germlings escape by phagolysosome-plasma membrane fusion without rupturing the host cells and penetrating the neighboring cells [29]. A recent report has shown that airway epithelial cells (A549) and bronchial epithelial cells (16HBE) internalize *A. fumigatus* conidia and digest the engulfed conidia inside the phagolysosome, and the killing is more in bronchial epithelial cells [30]. However, it is difficult to extrapolate these results to our data since the pathogen as well as the host are different in these studies.

Even though the efficiency of phagocytosis of the added conidia are similar [25], the number of differentially expressed genes in primary HCE cells was lower than RCB2280 cells under similar coculture experimental conditions. This data indicates that the primary cultures respond differently to fungal infection. It has been shown previously that the host cell’s immune response is more robust towards the germinated conidia compared to ungerminated conidia, which are covered by immunologically inert hydrophobins [31]. Taken together, our data imply that the primary culture prevented the immune activation by fungal conidia by preventing the germination, leading to the masking of activating signals on the conidial surface. The actual mechanism involved in the robust immune response against fungal infection by primary cells compared to cell line is not known and additional experiments are needed to explain this especially when the number of genes altered in infected primary cultures are less.

NanoString approach was used to analyze the immune response in air-liquid interface (ALI) cultures of primary HBECs upon exposure to *A. fumigatus* for 6 h, which showed 41 differentially expressed genes out of 730 immune genes [23]. We found 37 differentially expressed genes in corneal epithelial cells exposed to *A. flavus*. Morton et al., analyzed the expression of 117 genes on immune arrays and showed 16 differentially expressed genes in iDCs exposed to *A. fumigatus* [32]. Together, these results show that the host-pathogen interaction is complex and depends on several conditions, including the culture conditions, host cell type, and the fungal species used.

RNA seq approach has been used to study corneal epithelial cells [33, 34], but, to our knowledge, there are no studies on transcriptome analysis of corneal epithelial cells infected with fungi. Therefore, we compared the differentially expressed immune gene data with the gene expression profile of *A. fumigatus* infected bronchial epithelial cells [23]. This study also used NanoString analysis of mRNA but using ALI cultures for bronchial epithelial cells compared to single monolayer and detected 41 differentially expressed genes. Although these genes were found in our dataset, their expression level was not altered in the corneal epithelial cells upon *A. flavus* infection. This is not surprising since even in the same host cell (bronchial epithelium), *A. fumigatus* and *A. niger* alter the expression of different genes [35]. Immune-related genes IL8, CSF2, and NFKBIA, were increased in their expression level in *A. flavus* infected corneal epithelial cells, as has been shown in the case of *A. fumigatus* infected iDC [32]. IL8 is upregulated with the maximum fold change in RCB2280 cells infected with CI1123 in the NanoString data and ELISA. IL8 is a chemotactic factor that attracts immune cells, principally neutrophils, to the site of infection and is known to be secreted by several innate cells, including respiratory epithelial cells [36] and corneal epithelial cells upon *A. fumigatus* infection [37]. Several studies show IL8 is one of the inflammatory cytokines that remarkably elevated during fungal infection [20, 38, 39]. NFKBIA, which limits the immune response by inhibiting the nucleus translocation of NF-kB/REL complexes, was found to be upregulated in RCB2280 cells exposed to CI1123. In the case of *A. fumigatus* infection of A549 cells, NFKBIA is reported to be upregulated in *A. fumigatus* infected A549 cells at 8 h of infection as shown by Affymetrix gene microarray [40]; however, in a slightly different experimental system, A549 cells cultured in the transwell membrane and infected with *A. fumigatus* for 6 h, NFKBIA has been shown to be downregulated [41].

CSF2 (GM- GSF) is one of the highly up-regulated genes in RCB2280 cells infected with CI1698. CSF2 is crucial for the recruitment of monocytes and is involved in the neutrophil and monocyte antifungal activity against *A. fumigatus* in mice model of aspergillosis [42]. Primary airway epithelial cells cocultured with *A. fumigatus* for 6 h show an increase in the expression of CSF2 mRNA by microarray. However, the expression of CSF2 mRNA has not been altered in *A. fumigatus* cocultured with transformed bronchial epithelial cells (16HBE14o) (22). Our study found altered expression of CSF2 in only one clinical isolate infecting the corneal epithelial cell line. These results confirm that the variation between the outcome of fungal infection depends on the clinical isolate used as well as the host cell type.

Six genes were overlapped between RCB2280 cells infected with CI1698 and CI1123 including SLC2A1, TNFAIP3, ICAM1, RUNX1, ETS1, and IKZF3. SLC2A1 encodes a glucose-transporter in the mammalian blood-brain barrier. It has been shown to up-regulate in SV40 immortalized cells cultured in air-liquid interface compared to native corneal epithelial tissue [43] and reported to regulate the glycolytic metabolism in bacterial infection [44]. TNFAIP3 is involved in inflammatory response and is shown to upregulate in A549 exposed to *A. fumigatus* [20, 40]. ICAM1, a glycoprotein is expressed in human corneal epithelial cells from limbal explants and involved in cell motility. It is reported that the inflammatory cytokines modulate the ICAM1 expression in primary corneal epithelial cells and promote epithelial injury by binding to inflammatory cell receptors [45]. Runt-related transcription factor play a role in development of hematopoietic stem cells and blood cells. It has been shown to elevate in tuberculosis patients and plays a role in phagosome maturation [46]. Ikaros, a zinc finger transcription factor, IL-10 expression in CD4+ cells. It regulates the development and differentiation of CD4+ T helper cells and also regulates cytokines, cytokine receptors and cytokine signaling pathways [47, 48].

ETS1 gene was elevated upon infection by *A. flavus* clinical isolates. ETS1 is a transcription factor that regulates the expression of cytokine and chemokine genes [49]. Increased expression of IL6, IL8, IL23A, and IL19 is likely to be the result of altered ETS1 gene expression in infected epithelial cells. LGALS3, a gene that encodes galectin-3, a beta-galactoside binding protein, has been shown to reduce the fungal burden in case of *C. neoformans* [50] and *A. fumigatus* [51] in mice model. It is the only gene commonly altered in primary HCE cells in response to CI1698 and CI1123. Both the clinical isolates respond differently in immortalized cell line and primary HCE cells. This is in agreement with our previous report where it has been shown that the virulence of different clinical isolates of *A. flavus* is different in *G. mellonella* model [28].

PLAUR encoding the Urokinase receptor (uPAR) is involved in multiple functions [52] and produced by several cell types. Their role in wound healing, activation of neutrophils, and innate immune response imply that they are involved in the fungal infection as well. Viral infection of type II alveolar epithelial cell line A549 induces PLAU and PLAUR at early time points after infection [53]. PLAUR has been shown as one of the hub genes that strongly interact with other differentially expressed genes and reported as one of the key genes to be involved in Pneumonia caused by gram-positive bacteria [54]. The data implies that PLAUR is likely to be involved in fungal infections as well. PLAUR was found to be up-regulated in the epithelial cells infected with clinical isolate CI1698. Our results show that the PLAUR pathway of wound repair is common to lung epithelium and corneal epithelium.

Leukocyte immunoglobin-like receptors (LILRs) consists of 11 receptors with extracellular Ig-like domains, categorized into inhibitory (LILRB1-B5) and activating (LILRA1-A6 except A3) and reported to be expressed mostly in neutrophils [55]. LILRB2 suppress immune cell activity and mainly reported in viral, bacterial and parasite infection [56]. Recently it has been shown that LILRB2 is up-regulated in corneal tissues of bacterial keratitis. In this study, it was down-regulated in RCB2280 cells infected with CI1698 and primary cells infected with CI1123. CD6 and CD5 genes were highly upregulated genes in primary HCE cells stimulated with CI1698. CD5 and CD6 are the scavenger receptor, family members, and they recognize bacterial, fungal, viral, and parasite components [57]. CD5 has a protective role against fungal infection by recognizing and binding to fungal species via β-glucan [58], while CD6 recognizes and binds to bacterial LPS [59]. It will be interesting to examine the signaling event activated by this interaction in epithelial cells and their role in the antifungal response.

The comparison of immune gene expression between uninfected immortalized and un infected primary corneal epithelial cells showed many immune genes are abundantly expressed in primary corneal epithelial cells compared to immortalized cell line (data not shown). The response to *A. flavus* differs between the cells as there is a difference in the immune gene content in both the cells. Immortalized cells are diverged from primary cultures due to their genome instability [60]. So, the results were validated using primary cultures which are closer to the native condition.

There are other genes whose expression level is altered in fungal infection of corneal epithelium; however, their role in antifungal defense is not clear. Overall, the cumulative data from multiple sets of infection conditions presented in this report show that the genes involved in NF-kB signaling, TNF signaling, Th17 cell differentiation, B cell receptor signaling, complement, and coagulation cascade, cytokine-cytokine receptor interaction, and chemokine signaling were altered upon fungal infection of corneal epithelial cells. The results reported in this paper confirm that the epithelial cells are involved in antifungal defense actively by modulating defense pathways by cytokine production and inflammation. Further, our study also shows that the fungal strain and the host cell types show distinct mRNA expression profile changes during fungal infection.

## Conclusions

This is the first study examining the immune response of primary corneal epithelial cells and epithelial cell line exposed to *A. flavus* conidia from ocular clinical isolates. Our targeted transcriptome data using the NanoString approach showed that the gene expression profiles of primary cultures and cell lines of corneal epithelium are not identical. We detected that the genes associated with NF-kB signaling, TNF signaling, and Th17 signaling were upregulated, and the genes related to B cell receptor signaling and chemokine signaling were downregulated in one of the cells. These results show that the corneal epithelial cells could elaborate and influence immune and inflammatory responses during fungal infection.

## Supplementary Information

**Additional file 1: Table S1.** RNA transcript data for 595 genes profiled using NanoString’s Immunological Panel for RCB2280 cells infected with clinical isolates of *A. flavus.*

**Additional file 2: Table S2. **RNA transcript data for 595 genes profiled using NanoString’s Immunological Panel for primary HCE cells infected with clinical isolates of *A. flavus.*

**Additional file 3: Table S3. **Differentially expressed genes of RCB2280 cells and primary HCE cells infected with *A. flavus* CI1698 and CI1123.

## Data Availability

The datasets generated and analyzed are included in the article and additional files. NanoString RNA transcript data were deposited in the Gene Expression Omnibus (GEO) of NCBI under accession number GSE175824 with the following link: https://www.ncbi.nlm.nih.gov/geo/query/acc.cgi?acc=GSE175824.
